# Extraction of Peak Velocity Profiles from Doppler Echocardiography Using Image Processing

**DOI:** 10.3390/bioengineering6030064

**Published:** 2019-07-26

**Authors:** Amirtahà Taebi, Richard H. Sandler, Bahram Kakavand, Hansen A. Mansy

**Affiliations:** 1 Department of Biomedical Engineering, University of California Davis, One Shields Ave, Davis, CA 95616, USA; 2 Biomedical Acoustics Research Laboratory, University of Central Florida, 4000 Central Florida Blvd, Orlando, FL 32816, USA; 3 College of Medicine, University of Central Florida, 6850 Lake Nona Blvd, Orlando, FL 32827, USA; 4 Department of Cardiovascular Services, Nemours Children’s Hospital, 13535 Nemours Pky, Orlando, FL 32827, USA

**Keywords:** Doppler echocardiography, peak velocity profile, thresholding, image processing

## Abstract

The objective of this study is to extract positive and negative peak velocity profiles from Doppler echocardiographic images. These profiles are currently estimated using tedious manual approaches. Profiles can be used to establish realistic boundary conditions for computational hemodynamic studies and to estimate cardiac time intervals, which are of clinical utility. In the current study, digital image processing algorithms that rely on intensity calculations and two different thresholding methods were proposed and tested. Image intensity histograms were used to guide threshold choices, which were selected such that the resulting velocity profiles appropriately represent Doppler shift envelopes. The resulting peak velocity profiles contained artifacts in the form of sudden velocity changes and possible outliers. To reduce these artifacts, image smoothing using a moving average process was then implemented. Bland–Altman analysis suggested good agreement between the two thresholding methods. Artifacts decreased when image smoothing was performed. Results also suggested that one thresholding method tended to provide the lower limit (i.e., underestimate) of velocities, while the second tended to provide the velocity upper limit (i.e., overestimate). Combining estimates from both methods appeared to provide a smoother peak velocity profile estimate. The proposed automated approach may be useful for objective estimation of peak velocity profiles, which may be helpful for computational hemodynamic studies and may increase the efficiency of current clinical diagnostic tools.

## 1. Introduction

Cardiovascular disease is one of the leading causes of death in the world [1]. Doppler echocardiography is a common method used to provide quantitative and qualitative diagnostic information about blood flow velocity and direction. The measured velocity profiles are used in clinical settings to determine cardiac time intervals, which would provide useful information for various cardiovascular conditions [2,3,4,5]. Cardiac time intervals can be used to predict major cardiovascular events (MACE) including cardiac ischemia, heart failure, or cardiac death. These velocity profiles also have utility in research studies of cardiac physiology and hemodynamics. For example, profiles are useful in elucidating the relationship between physiological processes and seismocardiographic signals [6,7,8]. Profiles can also be used to develop realistic boundary conditions in computational fluid dynamics simulations of the heart and great vessels and can potentially lead to more accurate predictions of important local and systemic hemodynamic changes [9,10]. Peak flow velocities in different cardiac phases, including systolic, early, and late diastolic can also be measured from these velocity profiles [11].

Image processing has been used to analyze echocardiographic images including Doppler echocardiography [12,13,14,15,16,17]. In this paper, a study for the automatic extraction of temporal distribution of peak velocity profile from Doppler echocardiography images is presented. The Doppler principle is extremely helpful in evaluating the physiology and pathophysiology of the organ under investigation. Ultrasound machines generally acquire Doppler images of moving targets. However, tedious manual measurements are required to measure peak velocities and various cardiac time intervals. An algorithm, such as the one presented here, can automate these measurements and thereby improve workflow significantly. For example, on average, a complete pediatric echo consists of 20 manual measurements of Doppler velocities and time intervals. It becomes clear how efficient the acquisition would be with an automated algorithm. The proposed algorithm can be especially useful in measurements of aortic ejection time and aortic velocity time integral. These measurements correlate well with cardiac output and are obtained in every diagnostic study. This paper is organized as follows: Section 2 provides image acquisition methods as well as the proposed image processing algorithm; results are reported in Section 3; and conclusions are discussed in Section 4.

## 2. Materials and Methods

In Doppler echocardiography, the speed and direction of blood flow within the heart are determined noninvasively using the Doppler effect (a distinct advantage to invasive cardiac catheterization, expensive cardiac MRI, and without the radiation or dye reaction risks of CT angiography). In Doppler cardiography, the ultrasound beam should be parallel to the blood flow, which is a potential limitation of the method. Ultrasound machines make use of the Doppler shift principle to determine the direction and velocity of a structure (tissue or blood) based on the formula, ∆*f* = *f*_t_ − *f*_r_, where *f*_t_ and *f*_r_ are the transmitted and reflected frequency, respectively. The motion of the structure toward the ultrasound transducer results in compression of the reflected waves, reducing its period (i.e., negative ∆T, where T is the period) and thus increasing their frequency (i.e., negative ∆*f*). In the same manner, a positive ∆T would indicate an object moving away from the transducer. By convention, negative ∆T is displayed above the baseline and positive ∆T below the baseline. These principles are used routinely by ultrasound systems.

**Figure 1:**
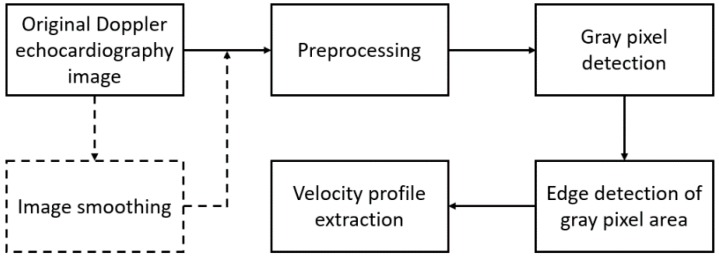
The proposed image processing steps for peak velocity profile extraction from Doppler echocardiography images. These steps are described in detail in the Section 2 of this paper. Dashed lines show the arbitrary steps.

In this study, an image processing algorithm was developed to extract peak velocity profiles from the echocardiographic images. The algorithm was based on identification and thresholding of gray pixels in the echocardiography images (Figure 1). Pulsed Doppler echocardiography videos were recorded from a cardiovascular ultrasound machine (Model: EPIQ 5, Philips, Netherlands) without moving or tilting the probe at a frame rate of 30 frames per second. The pulsed Doppler was acquired at sweep speed of 75 mm/s. The echocardiography video frames were then read as eight-bit unsigned integer images using MATLAB (R2015b, The MathWorks, Inc., Natick, MA, USA). Therefore, the color components of these images were integers in the range [0, 255]. Eight-bit images were used since they would provide enough information (e.g., tonal range and resolution) for estimating the peak velocity profiles in this study.

**Figure 2:**
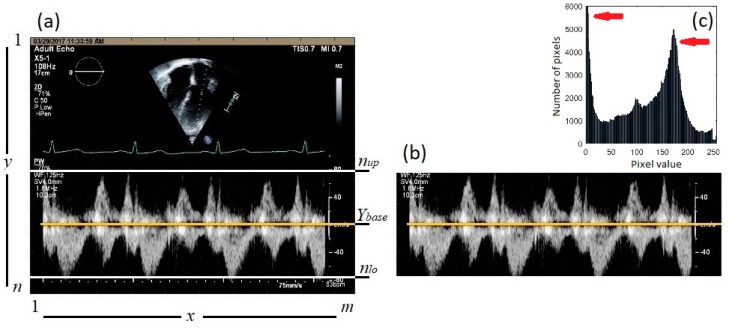
(**a**) A sample Doppler echocardiography image. The horizontal and vertical axes represent the number of pixels. The upper and lower borders of the region of interest (ROI) are marked with white solid lines. The baseline is shown with a yellow solid line. (**b**) The resulting image after removing the image portions outside the ROI. (**c**) Histogram of the ROI. The red arrows show the main peaks of the histogram which were used to determine the threshold values.

The images were considered as a scalar function *f* (*X*, *Y*), where *X* ∈ *x* = [1, …, *m*] and *Y* ∈ *y* = [1, …, *n*], and *m* and *n* are the number of pixels in the horizontal and vertical directions, respectively. Figure 2a shows a sample Doppler echocardiography image taken from the center of the mitral valve of a 55-year-old male with no history of heart disease. This image shows the four-chamber view of the heart, where the Doppler sample volume is placed in mitral valve inflow. The Doppler flow above the baseline depicts the inflow (from the left atrium to the left ventricle), and the Doppler flow below the baseline is the outflow (from the left ventricle toward the aorta). In Figure 2a, *m* and *n* are 1920 and 1080, respectively. The Doppler baseline (i.e., zero velocity) is shown as a yellow solid line at *Y_base_* = 790. Positive and negative shifts are displayed above and below this line and are indicative of blood flow toward and away from the transducer, respectively.

**Figure 3:**
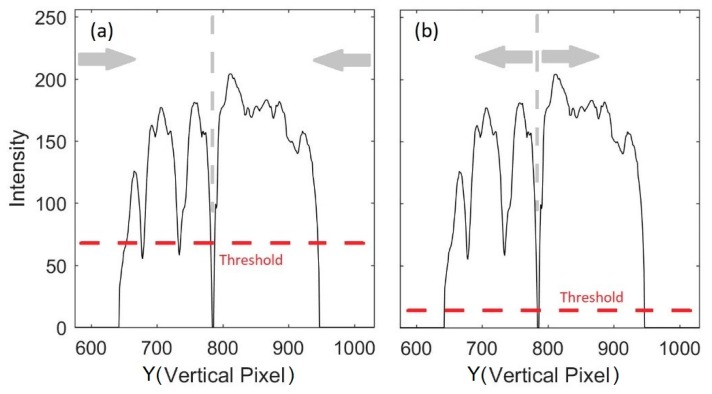
Gray pixels edge detection using (**a**) method 1 and (**b**) method 2. The averaged intensity is shown for (*X* = 450, *n_up_* < *y* < *n_lo_*). The dashed gray and red lines show the baseline and the threshold, respectively. For each method, the edge search direction is shown with gray arrows.

The proposed image processing algorithm for peak velocity profiles detection can be summarized in the following steps:

- Step 1: Load the raw echocardiographic image (such as the one shown in Figure 2a).
- Step 2: Define a region of interest (ROI) that encompasses the gray pixels containing the blood velocity information (1 < *x* < *m*, *n_up_* < *y* < *n_lo_*), where *n_up_* and *n_lo_* are the upper and lower borders of the ROI, respectively (Figure 2a) then remove pixels outside the ROI. For example, all pixels above *n_up_* = 575 and below *n_lo_* = 1030 in Figure 2a are removed. Figure 2b shows the resulting image. The histogram of the ROI is shown in Figure 2c. Once the ROI is defined, calculate the gray-scale intensity of pixels, *µ*, at pixel (*X* ∈ *x*, *Y* ∈ *y*).
- Step 3: Find the maximum intensity, *µ*, at each vertical line (i.e., at each *X* ∈ *x*) for *n_up_* < *y* < *n_lo_*, which was called *µ_max,X_*.
- Step 4: Detect gray pixel edges. Here, two different thresholding methods are attempted to detect the edge of the gray pixels in the ROI. In the first method (Figure 3a), edge search starts at the upper and lower borders of the ROI and moves toward the baseline. The upper and lower edges of the Doppler shift at each *X* are chosen as the smallest and largest *Y* ∈ (*n_up_* < *y* < *n_lo_*) such that *µ(X, Y)* > θ_1_ *µ_max,X_*, where θ_1_ is the threshold value, *µ(X, Y)* is the intensity of the pixel located at *(X, Y),* and *µ_max,X_* is the maximum intensity at time *X*. This method is designed to provide a lower limit of the velocity magnitude.

In the second method (Figure 3b), the edge search starts at the baseline and moves toward the ROI borders. The upper edge at each *X* is found as the smallest *Y* ∈ (*n_up_* < *y* < *Y_base_*) such that *µ(X, Y)* < θ_2_ *µ_max,X_*. The lower edge is calculated as the largest *Y* ∈ (*Y_base_* < *y* < *n_lo_*) such that *µ(X, Y)* < θ_2_ *µ_max,X_*. This method is designed to provide an upper limit of the velocity magnitude.

The intensity histograms of the ROI (Figure 2c) consisted of two main peaks around gray intensities of 0 and 175. These peaks are marked with red arrows in Figure 2c. The first peak corresponds to the black background. The threshold values for both methods, θ_1_ and θ_2_, were chosen to be between the two peaks such that they result in velocity profiles that agree with the expert opinion of two study specialists with more than 30 years of experience.

- Step 5: Construct the positive peak velocity profile by connecting the upper edges detected in Step 4 above. Then construct the negative peak velocity profile by connecting the lower edges. The pseudo-code for the proposed algorithm is provided in Algorithm 1. The code for the proposed algorithm in this paper is available online at: https://github.com/mirtatae/DopplerVelocityProfileExtraction.

**Algorithm 1.** Extraction of peak velocity profiles from Doppler echocardiographic images.1:**Input:** Doppler echo image, *img[n* × *m]*, upper and lower borders of ROI, *n_up_* and *n_lo_*2:**Output:** Upper and lower velocity profiles, *P_up_* and *P_lo_*3:*µ* = Intensity4:θ_1_ and θ_2_ = Thresholds5:Calculate *µ* at each pixel (*X*,*Y*) where *X* ∈ *x* = [1, …, *m*] and *Y* ∈ *y* = [1, …, *n*]6:
7:# first criteria8:**for** ∀ (*X*,*Y*) | *X* ∈ *x* and *n_up_* < *Y* < *n_lo_*9:   *µ_max,X_* ← max( *µ*(*X*,:) )10:   **if**
*µ*(*X*,*Y*) < θ_1_ × *µ_max,X_*11:     *µ*(*X*,*Y*) ← 012:   **end if**13:   *k* ← find all *Y* that *µ*(*X*,*Y*) > 014:   *P_1,up_* ← min(*k*)15:   *P_1,lo_* ← max(*k*)16:
**end for**
17:
18:# second criteria19:a = search window20:**for** ∀ *X* ∈ *x*21:   *µ_max,X,up_* ← max( *µ*(*X*,*Y_base_* − a:*Y_base_*) )22:   *i_up_* ← *Y* | *µ*(*X*,*Y*) = *µ_max,X,up_*23:   *k_up_* ← find all *Y* that *µ*(*X*, *n_up_*: *i_up_* + *Y_base_* − a) < θ_2_ × *µ_max,X,up_*24:   *P_2,up_* ← max(*k_up_*)25:
26:   *µ_max,X,lo_* ← max( *µ*(*X*,*Y_base_*:*Y_base_* + a) )27:   *i_lo_* ← *Y* | *µ*(*X*,*Y*) = *µ_max,X,lo_*28:   *k_lo_* ← find all *Y* that *µ*(*X*, *i_lo_* + *Y_base_* : *n_lo_*) < θ_2_ × *µ_max,X,lo_*29:   *P_2,lo_* ← min(*k_lo_*)30:
**end for**


The images were also smoothed by convolving with a square step kernel and the peak velocity profiles were estimated using the proposed algorithm. The pixels’ intensity was smoothed by a moving average process. Here, the smoothed image intensity, *µ_smooth_*, at (*X*, *Y*) was calculated as the mean intensity of a (2*p* + 1) × (2*q* + 1) rectangle centered at (*X*, *Y*):

(1) $$\mu_{smooth}\left(X,Y\right)=\sum_{j=Y-q}^{Y+q}\sum_{i=X-p}^{X+p}\mu \left(i,j\right)/\left(\left(2p+1\right)\left(2q+1\right)\right),$$

where *µ* was the image intensity before smoothing. The peak velocity profiles detected from the original and smoothed images were compared in order to investigate the effect of image smoothing. The E waves and A waves were then measured from the extracted profiles using the proposed algorithm and compared with the values manually calculated by the study specialists.

Edges of the Doppler shift (i.e., peak velocity profiles) were also detected using a standard edge detection method, Canny approximation. In this method, the gradient of the image intensity function is calculated using the derivative of a Gaussian filter [18]. The edges of the Doppler shift were then found by looking for local maxima of the gradient in the vertical direction. Canny approximation was selected since it is one of the edge detection methods that is less sensitive to noise [18]. The results were qualitatively compared with the results of our proposed algorithm.

## 3. Results and Discussion

**Figure 4:**
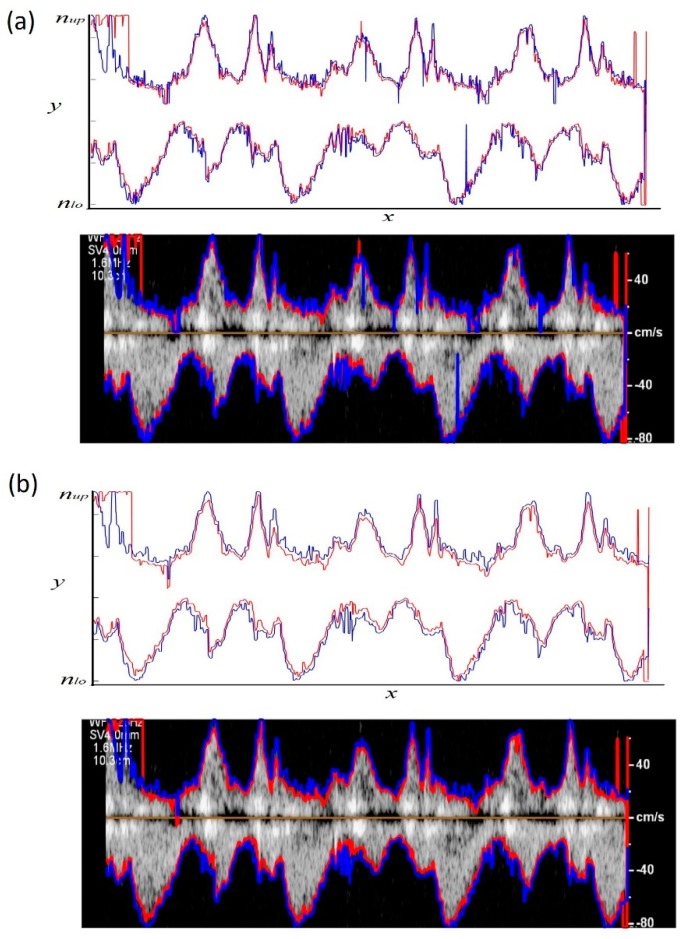
Peak velocity profiles extracted from a (**a**) raw and (**b**) smoothed Doppler echocardiographic image using the proposed thresholding: (red) Method 1 for θ_1_ = 0.3, and (blue) method 2 for θ_2_ = 0.05. Both positive and negative peak velocity profiles are shown. For the smoothed image, the velocity profiles are shown for *p* = *q* = 3 in Equation (2).

Two different thresholding methods were attempted to estimate the lower and upper limit of the peak velocity profile. Figure 4a shows the peak velocity profiles extracted from the Doppler image using both proposed thresholding methods. It can be seen that the lower limit of the peak velocity profile (red line in Figure 4a) was incorrectly estimated at the upper left edge of the ROI. In this portion of the ROI (1 < *x* < 200, 500 < *y* < 700 of the original image), there was some white text. This error resulted because the lower-limit thresholding method scanned the pixels at each vertical line starting from the borders of the ROI. Outside this ROI portion, the peak velocity profiles obtained from the two thresholding criteria showed general agreement, with the upper-limit velocity magnitude estimate being higher most of the time.

Although there was general agreement between the two estimates, both peak velocity profiles appeared to contain potential outliers and rapid velocity changes (as seen in Figure 4a). These results suggested adding a smoothing step to the algorithm as described in Section 2. Figure 4b shows the velocity profiles resulting from the smoothed Doppler image for both proposed thresholding methods. Here, the profiles appear to have less sudden changes compared to those of Figure 4a.

**Figure 5:**
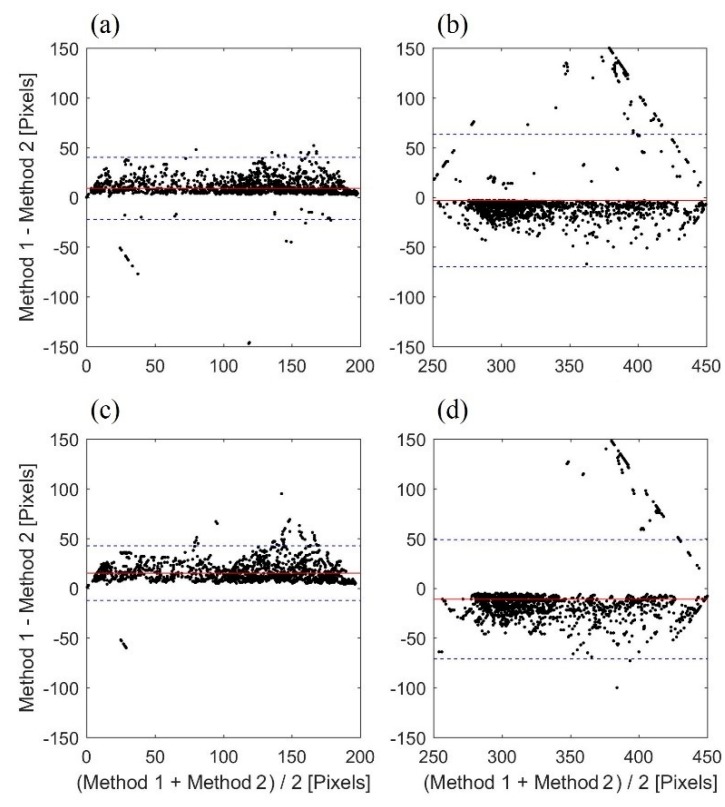
Bland–Altman analysis to assess the agreement between the two thresholding methods for (**a**), (**b**) negative and positive peak velocity profiles before smoothing, respectively; and (**c**), (**d**) negative and positive peak velocity profiles after smoothing, respectively. The solid line represents the mean value (i.e., bias) of the differences between two estimated profiles. The dashed lines show the 95% confidence interval (i.e., bias ± 1.96 SD). The unit of the horizontal and vertical axes is pixel, where each pixel corresponds to 0.34 cm/s.

To quantify agreement between the two thresholding methods (i.e., lower and upper velocity limits, respectively), Bland–Altman analysis was carried out. Results are shown in Figure 5a,b for the negative and positive peak velocity profiles extracted from the original image, respectively. Information is also shown for the profiles extracted from smoothed image in Figure 5c,d, respectively. Table 1 lists the bias and standard deviation of the difference between the two estimates. It can be seen that the standard deviation decreased with smoothing possibly due to random noise reduction. The bias increasing with smoothing is an indication of more separation between the upper and lower limits, which would be consistent with reduced noise in the estimates.

The agreement was higher for the negative velocities, which may be due to the reduced outliers that are represented by the points outside the confidence interval in Figure 5. These outliers are more prominent for the positive velocities. Outliers may be found from these plots and eliminated from the data to reduce errors in future studies.

**Figure 6:**
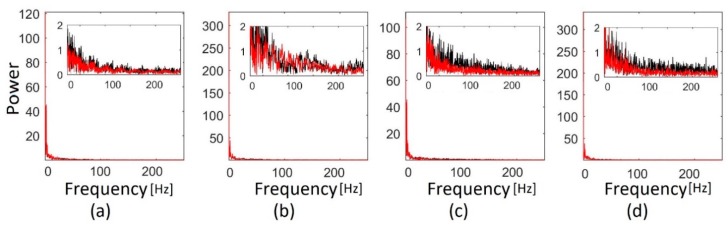
Fast Fourier transform of (**a**), (**b**) negative and positive peak velocity profiles using thresholding method 1, respectively; and (**c**), (**d**) negative and positive peak velocity profiles using thresholding method 2, respectively. Black and red lines show the spectra before and after smoothing, respectively. Each pixel in the Doppler image corresponded to 1.9 ms, which resulted in a sampling frequency of 532 Hz.

The spectra of the velocity profile of the non-smoothed and smoothed profiles are shown in Figure 6 and show significant reduction in high frequencies with smoothing. This further demonstrates the role of smoothing in reducing sudden velocity changes as expected.

**Figure 7:**
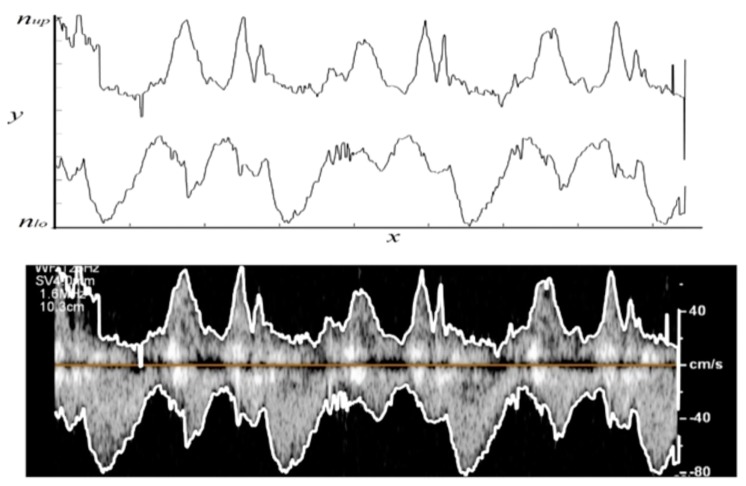
Averaged peak velocity profiles, *P_avg_*, extracted from smoothed Doppler echocardiography image. These profiles were calculated using Equations (2) and (3).

It is worth mentioning that the two proposed thresholding methods estimate the peak velocity differently and necessitate different threshold values. The first method starts searching for the peak velocity at the ROI borders and uses a relatively larger threshold to avoid artifacts at the borders. This method estimates a lower limit (underestimate) for the peak velocity magnitude. In contradistinction, the second method starts searching for the peak velocity from the baseline (i.e., zero velocity) and uses a smaller threshold, which would estimate an upper limit (overestimate) of the peak velocity profile. A combined method was also tested in the current study where the estimated velocity profiles from both methods are averaged (Figure 7). The averaged profiles, *P_avg_*, were calculated as:

(2) $$P_{avg,up}=\left(P_{1,up}+P_{2,up}\right)/2,$$

(3) $$P_{avg,lo}=\left(P_{1,lo}+P_{2,lo}\right)/2,$$

where *P_1,up_* and *P_1,lo_* are the upper and lower limits of the peak velocity profile from thresholding method 1, respectively. *P_2,up_* and *P_2,lo_* are the upper and lower limits of the peak velocity profile from thresholding method 2, respectively. 

The values of A and E waves are listed in Table 2. The last two columns show the R^2^ and Bland–Altman bias ± 1.96 SD between the study specialists and other methods. Results suggested that the bias between the specialist velocity measurements and the proposed method was 1.98 ± 11.20 cm/s for velocities in the range of about 65–75 cm/s.

**Figure 8:**
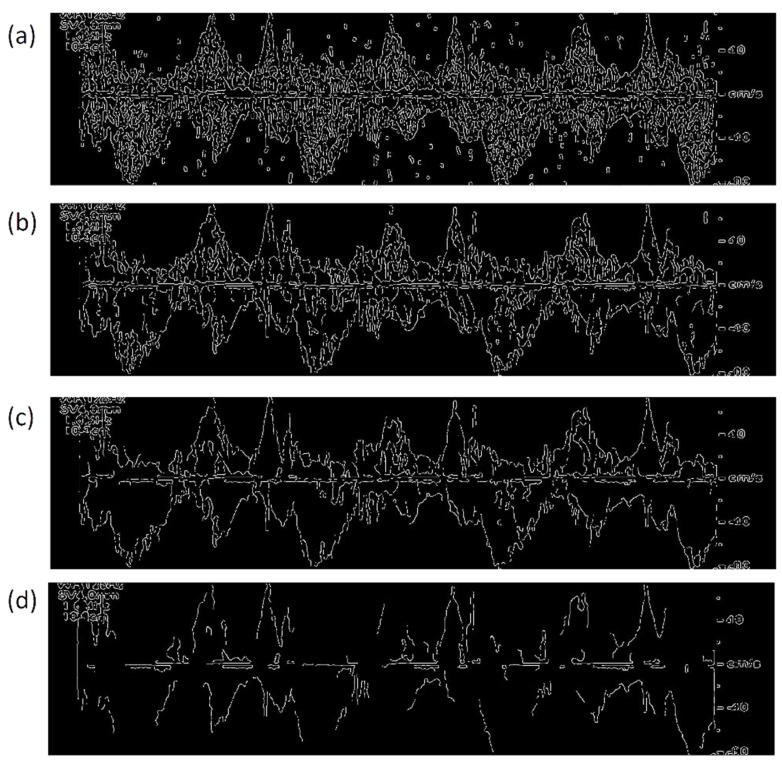
Edge detection in the vertical direction using Canny approximation employed on the smoothed image with a threshold value of (**a**) 0.05, (**b**) 0.20, (**c**) 0.30, and (**d**) 0.40.

Figure 8 shows the edge detection results obtained from Canny approximation applied on the smoothed image for four different thresholding values of 0.05, 0.20, 0.30, and 0.45. The Canny method with the smallest threshold (of 0.05) found the Doppler shift edges, but it also detected too many artifacts (e.g., other edges) both inside and outside of Doppler shift (Figure 8a). Figure 8b shows the detected edges using a higher threshold value of 0.20. Although the number of artifacts decreased, the algorithm still resulted in significant number of artifacts especially in the positive Doppler shift. An increased threshold (i.e., 0.30) was then used to reduce these extra unwanted edges (Figure 8c). As a result, the number of artifacts drastically decreased. However, there were still some artifacts around the baseline and Doppler shift borders. Figure 8d shows the results when a threshold of 0.40 was used. In this case, most artifacts were removed. However, due to a high threshold value, the algorithm missed many parts of the positive and negative peak velocity profiles resulting in disconnected profiles. Therefore, in this method, there is a compromise between avoiding artifacts and losing the peak velocity profile information. In contrast to the proposed method in this paper, the Canny method may find less or more than two edge points at each time (i.e., each vertical line). Finding more than two edge points at the same time is indicative of artifacts (such as artifacts seen in Figure 8a–c). Finding less than two points, indicates that the method did not properly estimate either one or both positive and negative Doppler shift envelopes. The proposed algorithm in this study, on the other hand, always estimates a lower and an upper edge in each vertical line. The proposed algorithm also resulted in continuous velocity profiles and less artifacts compared to the Canny method. In addition, it was simpler and less computationally expensive since it was only based on two simple thresholding operations. The proposed algorithm was also tested on 13 more images from the same subject. The results were consistent with the results shown in Figure 4 and Figure 7.

## 4. Limitations and Future Work

The proposed algorithm in this study was employed to detect peak velocity profiles from the images recorded by only one echo machine. In future studies, this algorithm should be tested on more subjects and different echocardiography machines. In addition, the performance of this algorithm was compared with the manual calculations of the study clinicians. The results were also qualitatively compared with a standard edge detection method. However, there are other algorithms for Doppler trace detection in literature [19,20,21] that outperform the Canny edge detector. The proposed algorithm should be compared against these algorithms.

## 5. Conclusions

An image processing algorithm based on thresholding was proposed to extract peak velocity profiles from Doppler echocardiography images. Two different thresholding methods were used to estimate the upper and lower limits of the peak velocity profiles. Doppler images were smoothed using the moving average process to improve the quality of the extracted velocity profiles. The upper and lower limits of the velocity profiles were also averaged, and a combining method was proposed. Bland−Altman analysis suggested that the bias between the specialist velocity measurements and the proposed method was 1.98 ± 11.20 cm/s. The velocity profiles extracted by the proposed method can be used in clinical and research studies including cardiac time interval estimations. It can also be useful in providing boundary conditions for computational cardiovascular hemodynamics studies. Future work would compare the estimates obtained in the current study with additional standard edge detection methods along with validating these profiles using more accurate velocity profiles from invasive cardiac catheterization. 

## Figures and Tables

**Table 1:** Statistical analysis results based on the Bland–Altman method. Each pixel corresponds to 0.34 cm/s.

	Difference between the Two Methods (Bias ± Margin of Error, 95% Confidence Interval) [cm/s]
Negative profile	3.10 ± 10.77
Positive profile	−1.12 ± 22.96
Negative profile after smoothing	5.20 ± 9.47
Positive profile after smoothing	−3.82 ± 20.62

**Table 2:** A waves and E waves (mean ± SD) calculated from the proposed algorithm as well as by the study clinicians.

Method	E Waves [cm/s]	A Waves [cm/s]	Bias ± 1.96 SD [cm/s]
Study specialists	62.33 ± 12.50	69.00 ± 7.94	
Raw image, method 1	67.93 ± 2.69	71.61 ± 1.96	4.10 ± 17.77
Raw image, method 2	66.78 ± 7.25	73.33 ± 1.11	4.39 ± 10.68
Smoothed image, method 1	61.38 ± 7.70	68.96 ± 3.10	−0.5 ± 12.11
Smoothed image, method 2	66.90 ± 7.08	73.33 ± 1.11	4.45 ± 10.82
Averaged profiles, *P_avg_*, Equation (2)	64.14 ± 7.30	71.15 ± 1.73	1.98 ± 11.20
